# The adolescent health network: A unique approach to sustained adolescent stakeholder engagement

**DOI:** 10.1017/cts.2024.567

**Published:** 2024-09-10

**Authors:** Deepa L. Sekhar, Alicia M. Hoke, Marwa Khan, Patricia L. Gordon, Erin K. Conahan, Jennifer L. Kraschnewski

**Affiliations:** 1 Department of Pediatrics, Penn State College of Medicine, Hershey, PA, USA; 2 School District of Lancaster, Lancaster, PA, USA; 3 Department of Medicine, Penn State College of Medicine, Hershey, PA, USA; 4 Department of Public Health Sciences, Penn State College of Medicine, Hershey, PA, USA

**Keywords:** Community-engaged research, patient-centered outcomes research, adolescent engagement, career development, adolescent health

## Abstract

At least 70% of premature adult deaths result from behaviors starting and reinforced in adolescence. The use of adolescent-centered outcomes and the necessity of creating space for the adolescent voice regarding research that directly impacts them is often overlooked. These omissions result in proposals and solutions that lack consideration of adolescent ingenuity, preferences, and needs. In 2021, Penn State PRO Wellness was awarded a Patient-Centered Outcomes Research Institute Engagement Award with the goal of addressing the gap in the inclusion of adolescents in research focused on teenage health. The resultant Adolescent Health Network (AHN) was developed in partnership with a stakeholder advisory board comprised of adolescents, parents, health researchers, and school staff. The AHN currently consists of 12 schools and 43 adolescents who have completed stakeholder training. For adolescents, the AHN simulates a school club or career enrichment activity with incoming freshmen replacing graduating seniors over time. For health researchers, the AHN provides rapid, easy access to a pool of adolescents with stakeholder training who are available to provide input on various aspects of a study from recruitment plans, to survey tools to dissemination strategies. This manuscript details the development, execution, and data from this novel program.

At least 70% of premature adult deaths result from behaviors starting and reinforced in adolescence [1]. Today’s adolescents face the dangers of noncommunicable diseases, e.g., obesity, physical inactivity, substance use, and mental disorders. These illnesses are further linked with health disparities including poverty, racism, environmental threats, educational inequalities, and their resulting adult morbidity [1,2].

Adolescents are increasingly engaging in autonomous healthcare decisions. Thus, it is of growing importance to engage them in the research and science that supports their health [3]. Yet, the use of adolescent-centered outcomes and the inclusion of the adolescent voice in research is often overlooked [1,4–7]. A 2021 study found less than one percent of studies collecting data from 12 to 18-year-olds included an adolescent advisory group [8]. Failing to engage adolescents creates a missed opportunity to educate our future generation of health researchers, academics, and healthcare providers.

There are unique challenges to engaging adolescent stakeholders. Teenagers grow out of adolescence, so after three to five years, these individuals no longer characterize a current teenage perspective [9,10]. As a vulnerable population, adolescent autonomy, compensation, and confidentiality can be difficult to navigate [10]. Yet, simple steps to facilitate youth engagement include meeting time selection with cognizance of school schedules, recognition in a way that is meaningful for youth (i.e., recommendation letter), and transparency in how youth feedback is used [11].

The Patient-Centered Outcomes Research Institute (PCORI), which funded this project, previously supported many studies involving adolescent stakeholders, but all were limited by lack of a sustainable framework for adolescent engagement [12–15]. Our primary objective was to develop and implement standard processes to build a sustainable framework to easily and quickly allow health researchers to connect with trained adolescent stakeholders for input during any phase of their research work from inception to dissemination. The Adolescent Health Network (AHN) was built on our Penn State PRO Wellness team’s history of partnering with Pennsylvania (PA) schools, specifically our Healthy Champions program [16].

This history of working with schools and our affiliation with an academic medical center created a natural bridge between adolescents and health researchers. Our aims were as follows: 1. Establish a stakeholder advisory board (SAB) of health researchers, school staff, parents, and adolescents to formalize policies and procedures for the AHN, 2. Build the AHN through Healthy Champions school contacts using the standardized engagement processes, and 3. Engage adolescent health researchers to utilize the network. Below are detailed our methods, program evaluation, challenges, and future plans.

## Methods

### Establishing the SAB

SAB members were recruited based on our Penn State PRO Wellness team contacts. PRO Wellness was initially established in 2003 by the PA Department of Health to fund healthy community activities in the state. The group was tasked with supporting PA schools to broaden their nutrition and physical activity offerings to meet national standards. Over its 20-year history the group has evolved, broadening its public health impact. In 2007, the group became a Center within Penn State, and in 2013 formally took on the PRO Wellness name (https://prowellness.childrens.pennstatehealth.org/).

Launched in 2013, Healthy Champions is one of PRO Wellness’ oldest programs. The program began with 57 schools but has enrolled an average of 500 PA schools annually in the past 5 years. Healthy Champions is primarily web-based; schools complete a baseline assessment on wellness practices in a variety of domains [16]. The assessment is scored, and schools are provided a variety of resources to support improvements curated by our PRO Wellness team [16]. In addition, the program supports a limited number of in-person school events promoting nutrition and physical activity [16].

Dr. Sekhar is the project lead and PRO Wellness Executive Director. In addition to Healthy Champions, PRO Wellness maintains partnerships with state agencies, schools, and community-based organizations via a wide variety of past and ongoing projects [16–19]. These relationships were leveraged to recruit a 12-member SAB composed of health researchers (three), school staff (three), parents (three), and adolescents (three).

For example, two adolescents were recruited based on a relationship with Aevidum, a student mental health organization [17,18]. Aevidum was a stakeholder in our Screening in High Schools to Identify, Evaluate, and Lower Depression (SHIELD) study [17]. Aevidum presented the SAB opportunity to student leaders, a couple of whom were interested in participating. In addition, during SHIELD, our team conducted parent and adolescent focus groups [20]. It is our standard practice to ask participants if they are interested in future opportunities. Another parent/adolescent pair was recruited via this mechanism. All adolescents were high school age (9^th^–12^th^ grades).

### SAB structure and training

Stakeholder engagement was facilitated by Ms. Hoke (coauthor), who served as the Community-Engagement Coordinator based on her past experience in this role [17]. The SAB met monthly for the initial six months of the project after which meetings transitioned to bimonthly. Meetings were held via web-based meeting platform. This allowed flexibility for adolescents to participate in nontraditional settings (e.g., car), for parents and school staff to participate during the work day without driving to our medical center campus, and was cognizant of COVID-19 restrictions. Materials were distributed approximately one week prior to the meeting and included action items for stakeholder completion prior to the meeting to increase engagement and discussion. Each meeting utilized multiple engagement modalities, including group discussion, polls, and breakout rooms with discussions focused on the perspectives of one type of stakeholder (e.g., adolescents, health researchers). The latter addressed commonly cited issues with power dynamics when engaging adults/professionals and youth together [11].

The initial SAB meetings included stakeholder training on the purpose, scope, and timelines of the project, along with completing FYREworks (adolescents, parents and school staff) and PCORI’s Research Fundamentals (health researchers as applicable) to standardize understanding of patient-centered outcomes research and community-based participatory research [15,21]. FYREworks (Family and Youth Research Education) was developed via a 2016 PCORI-funded Engagement Award to help youth and researchers create research partnerships [15]. Both trainings are web-based, self-paced modules [15,21].

### SAB-guided AHN development

**Figure 1.**
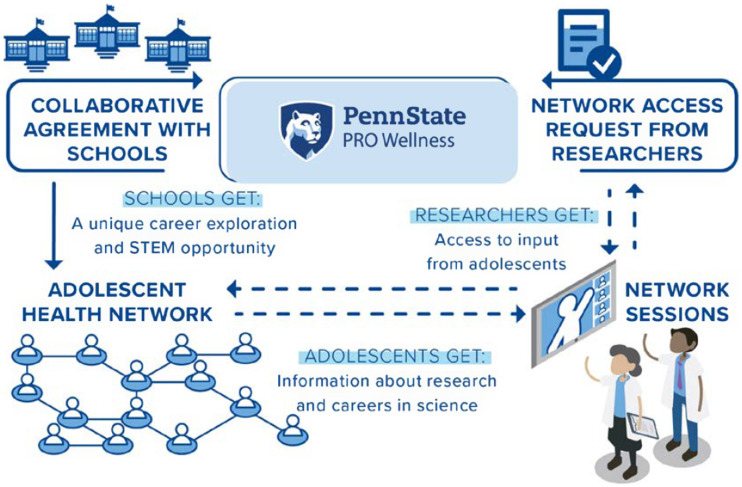
Overview of the adolescent health network components.

Once trained, the SAB-guided development of the processes and procedures to successfully launch and run the AHN (Figure 1). Stakeholders advised on format and wording for school and researcher recruitment materials, wording for a standard memorandum of understanding for partnering schools, and wording for the parent permission form. Adolescents gave feedback on training materials for peers, ultimately selecting FYREworks [15], and school staff and health researchers provided similar feedback for their peer training materials.

Stakeholders advised that adolescents serving on the AHN should be responsible for attending five to six sessions over the academic year to be considered members in good standing. In terms of compensation, the group felt it would be valuable to offer a letter confirming participation that could be used for volunteer hours, or college or career applications for adolescents in good standing. Adolescent stakeholders commented this would be more appreciated than monetary compensation, which would also limit sustainability.

The SAB helped to determine that each AHN session should be limited to the participating adolescents, two members of a research team, one school advisor, and one member of the PRO Wellness AHN team for introductions and technical assistance. Adolescent participation was capped at ten per session to allow for a variety of opinions, but not so many that individuals had limited opportunity to talk and participate. Thus, each AHN session was balanced between adults (four maximum) and adolescents to limit stifling adolescent conversation. The SAB agreed adolescents should participate with their cameras on but acknowledged technology limitations that would preclude this.

The SAB helped establish a grievance process for any issues arising during AHN participation from scheduling to sensitivity to researcher topics. Adolescents should first raise concerns with the school advisor, who could elevate the concern to the PRO Wellness AHN team. However, if the issue involved the PRO Wellness AHN team, recourse was provided for review via a separate third-party Penn State research group.

Throughout the two-year AHN development and implementation, stakeholders reviewed all evaluation materials and provided feedback on question wording and answer options. Stakeholders also participated in the development of a toolkit intended to provide a roadmap for health researchers at other academic institutions to develop their own AHN (https://prowellness.childrens.pennstatehealth.org/school/programs/adolescent-health-network/).

### School recruitment

Potential school partners were approached based on aforementioned PRO Wellness connections [16–20]. The AHN was presented to schools as a career enrichment, Science Technology Engineering and Math, and/or club opportunity. Schools were asked to identify an advisor, and these individuals represented a variety of school professionals, including guidance counselors, career counselors, and teachers. School advisors were asked to identify two to three adolescents for participation. Several responded with requests to include additional adolescents, enrolling four to five. Interested adolescents were tasked with completion of online permission forms with their parents/guardians and completion of FYREWorks [15]. School advisors were responsible to ensure adolescents completed training requirements, and to meet with adolescents to review and address any questions from the training.

School advisors were expected to volunteer to attend AHN sessions as their schedules allowed, such that at least one school advisor was present at each AHN session. While adolescents participated as stakeholders and not as research subjects, inclusion of a school advisor facilitated any needed follow-up in the event sensitive information was disclosed.

### Researcher recruitment

Once the AHN was populated with adolescents and school advisors, health researchers were recruited to utilize the resource. Recruitment occurred via DLS’ professional connections to pediatric health researchers and professional networks, through Penn State University networks (e.g., Clinical and Translational Science Institute [CTSI]), and through professional connections of the SAB health researchers. The PRO Wellness AHN team also promoted the AHN at relevant conferences including the American School Health Association annual meeting (7/2022), Action for Healthy Kids (11/2022), and the Association of Clinical and Translational Sciences meeting (4/2023). All presentations involved both the research team and stakeholder(s). Presentations were previewed by the SAB and adjusted based on SAB feedback.

### AHN session logistics

Interested health researchers submitted an online inquiry form, met with the PRO Wellness AHN team to discuss expectations and select a session date/time, and provided a 200-word lay summary highlighting why they were requesting adolescent feedback. AHN session registration opened to AHN adolescents and school advisors approximately four to six weeks prior to the session. In preparation for the AHN session, health researchers completed trainings and developed their discussion plan using the templates and resources provided by the PRO Wellness AHN team. Health researchers could provide up to 30 minutes of prereading, which was shared approximately one week in advance of the session.

Each 60-minute, web-based AHN session began with five to ten minutes of introductions and a brief recap by the researcher of what feedback was being requested. The next 40 minutes were facilitated by the health researcher addressing the topic and identifying questions. Health researchers were provided strategies to encourage adolescent participation. For example, health researchers were advised to start with easier questions. If one or two participants were dominating the discussion, health researchers were advised to call on individuals to offer an opinion. Health researchers were also advised to use the chat feature if participants were quiet.

The last ten minutes of the hour were reserved for health researchers to give back by sharing about their career path and how they became interested in their line of work. Adolescents also had the opportunity to ask questions. Health researchers received an audio-only session recording within 48 hours.

### Evaluation plan

Program evaluation was driven by two primary goals – 1. gather feedback to improve the function of the AHN processes and 2. understand how health researchers used the information gathered through engaging with the AHN (e.g., modify instruments, write a grant proposal). The latter was used to “close the feedback loop” with participating adolescents by sharing how their input was used by health researchers [22]. Thus, all participants (adolescents, school advisors, and health researchers) were invited to complete a brief-postsession electronic survey. Health researchers were contacted again 60 days after the session to better understand how feedback was utilized. Finally, adolescents and school advisors completed a mid-point and year-end program evaluation. All evaluation tools were developed and/or modified with the SAB and delivered as online surveys via REDCap, a secure, online tool for survey administration [23].

## Results

In the two-year development and implementation phase (2021−2023) 58 adolescents were nominated for AHN participation by school advisors from participating schools (*n* = 13). The 13 schools included two cyber schools, a technical school (57% economically disadvantaged and 22% Black student enrollment), and ten public high schools, six of which identified over 40% of students as economically disadvantaged and two with Latino student enrollment over 70% [24].

Fifty-three of those adolescents completed formal enrollment (i.e., parent permission, completed online training). Over the course of the program, ten additional adolescents discontinued participation either due to graduation (*n* = 4) or lack of interest/time to continue (*n* = 6). The latter includes students from one school that formally discontinued participation. The remaining 43 students from 12 schools are represented in the analyses and were invited to participate in the final program evaluation conducted in May 2023. Students were 72% female, 65% White, and 86% non-Hispanic (Table 1).

**Table 1.** Adolescent health network participant demographics

Demographic Information	*n*(%), Total *n* = 43
Gender	
Female	26 (72.2%)
Male	8 (22.2%)
Nonbinary	2 (5.6%)
Missing	7
Race	
American Indian or Alaskan Native	0 (0%)
Asian	3 (8.8%)
Black or African American	1 (2.9%)
Native Hawaiian or Pacific Islander	0 (0%)
White	22 (64.7%)
Other	8 (23.5%)
Missing	9
Ethnicity	
Hispanic or Latino	5 (13.9%)
Not Hispanic or Latino	31 (86.1%)
Missing	7
Graduation year	
2022	3 (8.3%)
2023	8 (22.2%)
2024	17 (47.2%)
2025	8 (22.2%)
Missing	7

There were 22 AHN sessions requested, and 21 were completed as one health researcher failed to schedule. There were 16 unique health researchers from eight unique academic institutions. Three health researchers requested multiple sessions. Requests generally fell into the following categories: review of study recruitment materials (e.g., word choice, clarity), review of study materials (e.g., surveys, focus group guides, and consent forms), requests to review study methods (e.g., recruitment plan, compensation), and requests to provide advice on dissemination of results. Researcher topics varied widely, e.g., mental health screening, school wellness policies, social media use, and adolescent-parent relationships.

### Postsession evaluations

A total of 76 postsession surveys were completed by adolescents across the 21 completed AHN sessions; an average postsession survey response rate of 59% per session. A total of 14 postsession surveys were completed by school advisors for the 21 sessions, with a response rate of 67%. Nearly every adolescent and school advisor felt health researchers spoke about the topic using understandable language (99% and 100%, respectively, Table 2). The vast majority of adolescents and school advisors felt the discussion was comfortable (93% and 86%, respectively). Compared to school advisors, fewer adolescents found discussing the health researcher’s career path useful (75% vs. 100%).

**Table 2.** Forty-eight-hour postsession student and school staff advisor feedback

Evaluation questions	*n*	Disagree Total (%)	Neutral Total (%)	Agree Total (%)
The researcher spoke about the topic using words and phrases I understood				
Adolescent	76	0 (0%)	1 (1.3%)	75 (98.7%)
Advisor	14	0 (0%)	0 (0%)	14 (100%)
The session started and ended on time				
Adolescent	76	0 (0%)	4 (5.3%)	72 (94.7%)
Advisor	14	0 (0%)	0 (0%)	14 (100%)
The discussion of the health researcher’s career path was useful				
Adolescent	74^*^	0 (0%)	17 (22.4%)	57 (75%)
Advisor	13^**^	0 (0%)	0 (0%)	13 (100%)
Most students participated in the discussion				
Adolescent	76	2 (2.6%)	8 (10.5%)	66 (86.8%)
Advisor	14	2 (14.3%)	0 (%0)	12 (85.7%)
The format of the session was appropriate for the topic of discussion				
Adolescent	76	0 (0%)	2(2.6%)	74 (97.4%)
Advisor	14	0 (0%)	0 (0%)	14 (100%)
The information shared about the project prepared me for the discussion				
Adolescent	76	2 (2.6%)	6 (7.9%)	68 (89.5%)
Advisor	12^*^	(0)	2 (14.3%)	10 (71.4%)
The discussion was comfortable				
Adolescent	76	0 (0%)	5 (6.6%)	71 (93.4%)
Advisor	14	0 (0%)	2 (14.3%)	12 (85.7%)
There was adequate time between when a session sign-up was made available and the actual session				
Adolescent	76	0 (0%)	0 (0%)	76 (100%)
Advisor	13^***^	0 (0%)	0 (0%)	13 (100%)

* N/a was an option for this question selected by 2 individuals.

** N/a was an option for this question selected by 1 individual.

*** 1 missing response.

Approximately 86% of health researchers completed a 48-hour postsession survey (*n* = 18/21). Health researchers all agreed (18/18 respondents) that the feedback provided by students was helpful, and the meeting format was appropriate for the research topic. Training and participation in a session modestly improved the participating health researchers’ confidence to engage in community-engaged research. Approximately 11% (*n* = 2/18) of health researchers identified as “not at all confident” prior to training and 6% (*n* = 1/18) prior to participating in a session. This decreased to 6% (*n* = 1/18) and 0% after training or participating in a session, respectively.

### Health researcher 60-day survey

Eleven out of 21 health researchers completed the 60-day-postsession survey (52%). All (100%) responding health researchers would recommend the AHN to a colleague. The majority (9/11, 82%) had already modified their work based on the session, with the remaining two indicating they intended to do so in the next six months. Over half (6/11, 55%) had already applied for funding for the project discussed. Fewer health researchers made use of the session audio recording (3/11, 27%), and 46% (5/11) indicated they did not intend to use the recording to review points made during the session.

### Adolescent and school advisor final program evaluations

Of the 43 students eligible for participation in May 2023, 24 (56%) completed the final program evaluation. Of the 24 respondents, 46% felt the program was less work than expected, and 54% indicated it was the amount of work expected. Most (83%) would have signed up for a few more sessions had they been offered at different times. All respondents reported understanding the difference between a stakeholder and a research subject. The majority (96%) would recommend joining the AHN to their peers. Students described several barriers to participation in AHN sessions including competing schedules during the school day and after school (due to extracurricular activities).

Participating adolescents (96%, *n* = 21/22) reported agreement with their ability to represent a youth perspective while providing feedback to the health researchers and felt that at least some of the interactions with the health researchers sparked their interest in a research career (91%, *n* = 20/22; Table 3). Both training and participation in a session improved the participating adolescents’ confidence to participate in the sessions to guide health researchers. All adolescents identified as either “moderately confident” (41%, 14%) or “very confident” (59%, 86%) after completing training or participating in a session, respectively.

**Table 3.** Adolescent final evaluation (*N* = 22)*

Evaluation questions	Disagree *n*(%)	Sometimes *n*(%)	Agree *n*(%)	
I can easily locate the zoom link for each session.	1 (4.5%)	7 (31.8%)	14 (63.6%)	
I can easily access the zoom platform to participate in each session.	1 (4.5%)	3 (13.6%)	18 (81.8%)	
I attend sessions with my camera on.	1 (4.5%)	0 (0%)	21 (95.5%)	
I am able to represent a youth perspective while providing feedback to the health researchers.	0 (0%)	1 (4.5%)	21 (95.5%)	
Interaction with the health researchers sparked my interest in a research career.	2 (9.1%)	10 (45.5%)	10 (45.5%)	
I complete the feedback survey after each session.	1 (4.5%)	5 (22.7%)	16 (72.7%)	
	Not at all useful	Somewhat useful	Very useful	N/A – did not use
FYREWorks training modules (i.e., stakeholder training)	1 (4.5%)	14 (63.6%)	6 (27.3%)	1 (4.5%)
Mock session video	0 (0%)	6 (27.3%)	12 (54.5%)	4 (18.2%)
Network logistics information (on shared resource portal)	1 (4.5%)	5 (22.7%)	10 (45.5%)	6(27.3%)
Meeting(s) with your school’s project advisor	0 (0%)	4 (18.2%)	10 (45.5%)	8 (36.4%)

* Data are presented on 22 student respondents due to 2 not having participated in any sessions and thus being unable to answer all questions.

School advisors had positive feedback about the program. Most (10/12, 91%) felt the session topics were appropriate for their students. The majority felt the FYREworks stakeholder training, mock session video, and network logistics information were very useful (82%, 73%, and 75%, respectively).

## Discussion

Building the AHN created a unique opportunity to facilitate the connection between adolescent stakeholders and health researchers to improve research work. In an era of overwhelming access to often conflicting scientific information [25,26], engaging youth to better understand the scientific process and develop their skills as consumers of the scientific literature is of critical importance. Participating adolescents expressed increased confidence to share their opinions with health researchers and interest in future research careers. School advisors also commented on the great career conversations between health researchers and adolescents. Prior work has focused on volunteer opportunities and clinical and clerical experience to spark adolescent interest in healthcare and research, especially for underrepresented minorities (e.g., Reach One Each One Program [ROEO]) [27,28]. Of participating ROEO seniors enrolled in college, 21/24 (88%) elected a health science degree [28]. Similarly, 91% of AHN participants completing the final evaluation expressed interest in a health research career. To our knowledge, this is the first program to link an adolescent stakeholder experience to interest in future healthcare and research careers. Yet, the project team encountered challenges, discussed below, including how best to effectively sustain the AHN at the end of the funding period.

Students initially nominated for participation by school advisors were “superstars,” e.g., high achievers and active participants; 36% identified as non-White. It is challenging to bring more reticent, underrepresented voices to the table [11,29]. An AHN participant suggested “bring a friend” as a potential solution. AHN adolescents would bring a more reluctant peer to a session for a clearer understanding of how the program runs.

Additional approaches to diversify participants include asking school advisors to consider a broader group of students or using an open application process. In addition, the team can broaden the demographics of schools approached to join the AHN. Scheduling the AHN sessions was another barrier to diverse participation. Adolescents in sports, school clubs, or working due to financial needs had commitments at the end of the school day. The team attempted to vary session times based on participant feedback, but it remained a challenge to find times that worked well for everyone.

There are pros and cons to the use of school-based networks. The school structure allows incoming freshmen to replace graduating seniors similar to other school clubs and activities. Thus, the pool of trained adolescent stakeholders is “renewable.” Yet, working with schools introduces limitations. Most school advisors did not want to participate in evening AHN sessions, and student recruitment was at the discretion of the school advisors. Communication was sometimes challenging if school email servers blocked messages from external senders. Once this was discovered alternate emails and text message reminders were added.

An additional unanticipated difficulty was connecting with health researchers. There is a recognized gap in patient and stakeholder engagement, specifically among pediatric health researchers [8,30]. Many health researchers thought the AHN was a creative, valuable concept, but struggled with how to realistically apply adolescent input to their work. If given concrete examples, e.g., adolescents could provide input on the wording of a survey or guidance on study recruitment materials, health researchers were more readily able to make the connection. Due to this difficulty, general recruitment methods (newsletters) were less successful than an individualized, targeted approach during which the PI (Sekhar) could actively explore the concept of community-engaged research with the health researcher.

Finally, anticipating the end of the project funding period, the PRO Wellness team streamlined AHN processes and evaluations and determined the minimum degree of staffing to maintain the AHN in its current form. Since submission of this manuscript, the AHN successfully received funding through our Penn State CTSI Community-Engaged Research Core. This is very exciting as the team hopes to continue the AHN and work toward addressing the aforementioned challenges in diversity of adolescent participants and researcher recruitment.

In summary, the AHN is a unique approach to address adolescent stakeholder engagement in research. Evaluation demonstrated benefits for both adolescents and health researchers; the program remains open for consultation. With the current CTSI funding, the team will continue to grow and develop the AHN to best support quality adolescent health research.
