# The adolescent health network: A unique approach to sustained adolescent stakeholder engagement – CORRIGENDUM

**DOI:** 10.1017/cts.2025.10060

**Published:** 2025-06-02

**Authors:** Deepa L. Sekhar, Alicia M. Hoke, Marwa Khan, Patricia L. Gordon, Erin K. Conahan, Jennifer L. Kraschnewski

**Affiliations:** 1 Department of Pediatrics, Penn State College of Medicine, Hershey, PA, USA; 2 School District of Lancaster, Lancaster, PA, USA; 3 Department of Medicine, Penn State College of Medicine, Hershey, PA, USA; 4 Department of Public Health Sciences, Penn State College of Medicine, Hershey, PA, USA

This notice is to advise that some errors were present in the references in the original article. The description of these errors can be seen below:

1) References 14 and 15 were reversed.

Reference 14. Listed as Bevans should be Connolly (Connolly M. Motor City School Health Collaborative. Patient-Centered Outcomes Research Institute website. Updated March 27, 2020. Accessed January 6, 2020. https://www.pcori.org/research-results/2017/motor-city-school-health-collaborative)

Reference 15. Should be Bevans (Bevans K. Family and Youth Research Education (FYREworks) Trainings and Resources for Research Partnerships Involving Adolescents. Patient-Centered Outcomes Research Institute website. Updated September 3, 2020. Accessed January 6, 2021. https://www.pcori.org/research-results/2016/family-and-youth-research-education-fyreworks-trainings-and-resources-research.)

Reference 8 should be replaced with Sellars E, Pavarini G, Michelson D, Creswell C, Fazel M. Young people’s advisory groups in health research: scoping review and mapping of practices. *Arch Dis Child*. 2021;106(7):698-704. doi: 10.1136/archdischild-2020-320452
